# Editorial: Insights in ADHD: 2023

**DOI:** 10.3389/fpsyt.2024.1490699

**Published:** 2024-09-17

**Authors:** Susan dosReis

**Affiliations:** Department of Practice, Sciences, and Health Outcomes Research, University of Maryland School of Pharmacy, Baltimore, MD, United States

**Keywords:** ADHD, pediatrics and adults, clinical practice, emotion dysregulation, biological mechanism, treatment, diagnosis

The Research Topic on Attention-Deficit/Hyperactivity Disorder (ADHD) entitled, “Insights in ADHD,” comprises seven papers that encompass a range of evidence from biological mechanisms to clinical practice guidelines. The Research Topic brings together papers that offer a global perspective on insights into ADHD. The papers address prevalence and clinical management, dose optimization, and the neurocognitive mechanisms. Two studies discuss the association between ADHD and external factors. The seven papers are briefly discussed within the broader thematic topics covered in the Insights in ADHD Research Topic.

Takaesu et al. conducted a cross-sectional study using the Japanese health insurance claims data to investigate the prevalence of somatic disorders among adults with and without a history of ADHD. The control group without a history of ADHD was matched 5:1 to adults with ADHD. The somatic disorders studied were major common chronic conditions, such as type 2 diabetes mellitus, cardiovascular disease and liver disease. They further stratified their cohort by presence of mental disorders. The investigators found a higher prevalence of somatic disorders among adults with ADHD, and among those who also had a mental disorder. The study brings to light a higher burden of illness among adults with ADHD. Trends show an increase in adult ADHD as stimulant use has increased from 2016 to 2021 in US adults age 40 or older (1). Coupled with the onset of many major chronic conditions with age, the study findings have important implications for excessive burden of illness for a growing segment of the population with ADHD.

The study by Öğütlü et al. describes the Turkish DETECT consensus report to assist clinician management of ADHD among Turkish youth. This review provides an extensive discussion of the etiology of ADHD, including genetic, neurobiological, neurophysiological, and neuropsychological factors. The diagnostic criteria and standard tools to assist in the diagnostic evaluation are reviewed but they also emphasize the importance of detailed history. The critical need for a differential diagnosis to rule out conditions that may resemble ADHD and a thorough assessment of the youth’s development or comorbid conditions are reviewed in detail. They conclude with a review of pharmacologic and non-pharmacologic treatment interventions. This paper brings to the forefrong the multiple complex factors contributing to ADHD, and for which a comprehensive differential diagnosis is critical to determine the best treatment.

In the United States, Cutler et al. report on a 21-day dose optimization open-label study in a multi-center, double-blind, randomized placebo-controlled laboratory classroom. The study sample included children ages 6 to 12 with ADHD. Serdexmethylphenidate/dexmethylphenidate was initiated at 39.2/7.8 mg and could be titrated for maximal efficacy to one of three doses: 26.1/5.2 mg, 39.2/7.8 mg, or 52.3/10.4 mg. The dose optimization open label study found that the 80% of children with dose optimized to 39.2/7.8mg or 26.1/5.2mg had ‗ 50% response rate. Importantly, significant improvements were observed as early as 7 days after baseline. The benefits of lower doses to achieve clinical improvement has been noted in earlier studies (2, 3). While not all individuals will respond to lower doses, an adequate trial of an effective lower dose should be used prior dose escalation.

The cross-sectional study by Glans et al. investigates whether body modifications are indicators of subclinical ADHD. The authors posit that tattoos and body piercings are types of impulsive behaviors and may be indicative of ADHD. The sample of Swedish adults ages 18 to 65 years old who had not been diagnosed with ADHD completed a questionnaire that measured ADHD symptoms, body modifications, and demographic information. The investigators report that body modification of any type was associated with higher scores on the total and hyperactivity/impulsivity subscale of the WHO Adult ADHD Self-Report Scale. Piercing was associated with medium to large effect sizes, and especially with increasing number of piercings. Further study is warranted to better understand the implications of body modifications in the context of ADHD.

Nowak et al. conducted a case-control trial of age- and sex-matched soccer players and individuals from local universities in the United States. Their study was designed to assess the influence of ADHD on neuro-ophthalmologic function and brain-derived blood biomarkers following acute sub-concussive head impacts. This research is based on the investigators’ prior work showing that ADHD reduces resiliency to trauma induced brain injury (4). The researchers found that ADHD may be related to impaired neuro-ophthalmologic response to sub-concussion. The study offers some important clinical implications given the vulnerability to cellular and neuro-ophthalmologic deficits associated with ADHD. While the results in and of themselves are not conclusive, this study provides a basis from which future studies can build upon to better understand neurological measures associated with ADHD.

Isaac et al. from Chile examined arousal dysregulation and executive dysfunction in ADHD. The authors address the etiological basis for ADHD, which has heterogenous biological and physiological characteristics that underpin the observed variability in behavioral and cognitive symptoms. Specifically, they discuss the theoretical constructs of executive dysfunction and state regulation through the lens of executive function theory and state regulation theory as the foundation for an integrative framework. The authors call for additional research on indicators of symptoms that will better differentiate subgroup variability in ADHD. Such knowledge could contribute to greater accuracy in a differential diagnosis, and ultimately aid in more precise and targeted interventions.

Marques et al. conducted a study in Portugal to investigate whether emotion dysregulation and depressive symptoms are mediators of inhibitory control and impulsive behaviors in ADHD. Their study included children ages 6-10, of which 38 had a diagnosis of ADHD and 34 were ‘typically developing,’ or absent of ADHD. The authors constructed an emotional dysregulation factor using principal axis factoring. They reported that children with ADHD had higher scores, i.e., greater impairment, than typically developing children. Emotional dysregulation mediated the association between problems with inhibitory control and aggressive behavior, explaining 61.8% of the variance in aggressive behavior. The authors also report a direct relationship between inhibitory control difficulties and emotion dysregulation, which in turn leads to greater depressive symptoms that then results in more aggressive behavior. Disruptive behaviors among youth with ADHD contributes to more complex medication regimens, often involving an antipsychotic (5, 6). A better understanding of the mechanistic pathways can inform targeted interventions that could potentially reduce the need for complex, and sometimes dangerous psychotropic medication (7–9).

Globally, ADHD affects 7.6% of children ages 12 or younger and 5.6% of adolescents ages 13-18 (10). It is one of the most extensively studied neurodevelopmental disorder, and pharmacologic and non-pharmacologic treatment interventions are supported by decades of evidence-based research. Nonetheless, there remain many questions about the biological mechanisms, the role of emotion regulation, and the extent of behavioral risk factors that may impact or may be the consequence of ADHD. This Research Topic brings to light the global presence of ADHD and the critical need for scientific investigations that can fill the knowledge gaps.
